# Glassfiber Post: An Alternative for Restoring Grossly Decayed Primary Incisors

**DOI:** 10.5005/jp-journals-10005-1158

**Published:** 2012-08-08

**Authors:** Manjul Mehra, Rashu Grover

**Affiliations:** Senior Lecturer, Department of Pedodontics and Preventive Dentistry Sri Guru Ram Dass Institute of Dental Sciences and Research Amritsar, Punjab, India, e-mail: mehramanjul@yahoo.co.in; Senior Lecturer, Department of Pedodontics and Preventive Dentistry Sri Guru Ram Dass Institute of Dental Sciences and Research, Amritsar Punjab, India

**Keywords:** Primary incisors, Glassfiber posts

## Abstract

Restoration of primary incisors, which have been severely damaged by rampant caries or trauma, is a difficult task for the pediatric dentist. With the introduction of new adhesive systems and restorative materials, alternative approaches for treating these teeth have been proposed. This paper discusses the restoration of carious primary maxillary incisors using composite resin restoration reinforced with fiberglass post. Two case reports are presented here to describe the procedure. Over a 1 year period, the crowns have demonstrated good retention and esthetic results.

**How to cite this article:** Mehra M, Grover R. Glassfiber Post: An Alternative for Restoring Grossly Decayed Primary Incisors. Int J Clin Pediatr Dent 2012;5(2):159-162.

## INTRODUCTION

Caries has affected human race since prehistoric times and still is one of the most prevalent oral diseases of modern times. One of the forms of caries affecting early childhood is rampant caries which grossly involves the primary anterior teeth to the extent that they usually need endodontic treatment.^1^ Another major factor which is concern for the dentist is trauma.^2^ The irony of the situation is that these patients usually approach the dentist only when the teeth are grossly broken down and merely the root stumps are left. In the past, the most expedient treatment was to remove the involved teeth. This treatment was justified on the basis that the permanent teeth would eventually replace the extracted ones. However, the importance of preserving the integrity of primary dentition until the appropriate exfoliation time is well recognized. The consequences of premature loss of primary teeth are well known namely the loss of vertical dimension of occlusion, space maintenance problems, phonetic alterations, development of parafunctional habits and psychological problem.^3,4^

Due to the development of restorative materials and new restorative technique in dentistry, treatment of primary maxillary incisor teeth cannot be neglected. In cases with severe crown destruction and where merely the root stumps are left, endodontically treatment associated with the use of intracanal posts become necessary prior to restoration of the crowns. This allows us to restore normal masticatory function, phonetics and esthetics to the child.^5,6^

The various root canal posts used in pediatric dentistry are orthodontic thread in the shape of alpha or gamma,^7^ the metallic posts with macro retention,^8^ composites posts,^2^ biological posts,^3^ and the fiberglass post.^9^

Recently dental manufacturers have developed glass fiber post to repair grossly decayed teeth. These are composed of unidirectional glass fibers embedded in a resin matrix that strengthens the dowels without compromising the modulus of elasticity. Another advantage of glass fibers is that they distribute stresses over a broad surface area, increasing the load threshold.^9^ This paper discusses the restoration of carious primary maxillary incisors using composite resin restoration reinforced with fiberglass post. Two case reports are presented here to describe the procedure.

## CASE REPORTS

### Case 1

**Fig. 1:**
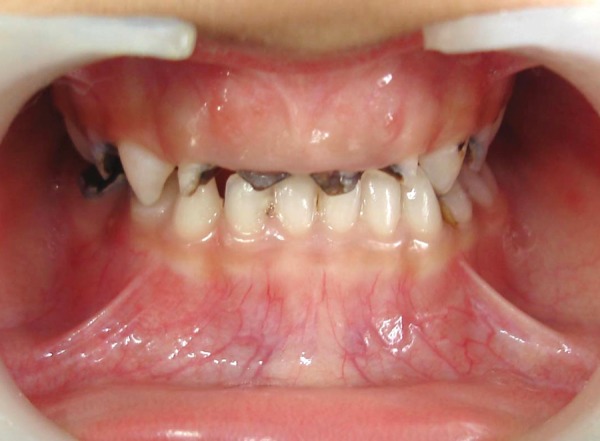
Preoperative (case 1)

A 3-year 4-month-old male child presented with the chief complaint of decayed upper anterior teeth. Patient's medical history was noncontributory. On examination complete set of primary dentition was present, but maxillary incisors, molars and mandibular molars were severely destroyed due to caries. Clinically the root stumps of maxillary anterior were found to be firm, with an extension of the remaining crown of less than 1mm above the gingival margin (Fig. 1). An intraoral periapical radiograph of these teeth showed totally intact roots and normal development of permanent successors. The child's parents were informed about the treatment plan. Pulpotomy and restoration with stainless steel crown in relation to 54, 64 and pulpectomy and restoration with stainless steel crown for 84, 85 were carried out and it was decided to restore maxillary anterior with fiberglass post as described below.

### Endodontic Treatment

**Fig. 2:**
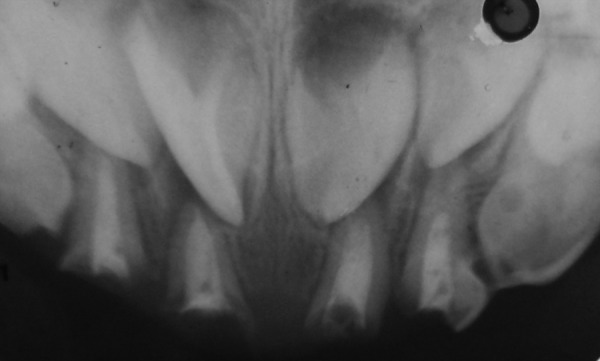
Postoperative radiograph

Caries removal has to be done carefully being as conservative as possible, keeping intact hard dentin. Endodontic treatment of the retained root stumps was carried out using zinic oxide eugenol as the obturating material (Fig. 2). The pulpal treatment will not be discussed in detail here as this is not the aim of this article.

### Creating Space for Post

After completion of the pulpectomy of maxillary anterior, to a suitable depth, a 4 mm length of the coronal portion of the root filling was removed (2-3 mm below the CEJ). This could be done with the same bur that removes pulp tissue or with the special drill that comes in the fiber core kit. A thin layer of fast-setting glass ionomer cement was then condensed over the obturating material to act as a barrier between the obturating material and the resin restoration to prevent inference with the setting process of the composite resin.

### Post Insertion

**Fig. 3:**
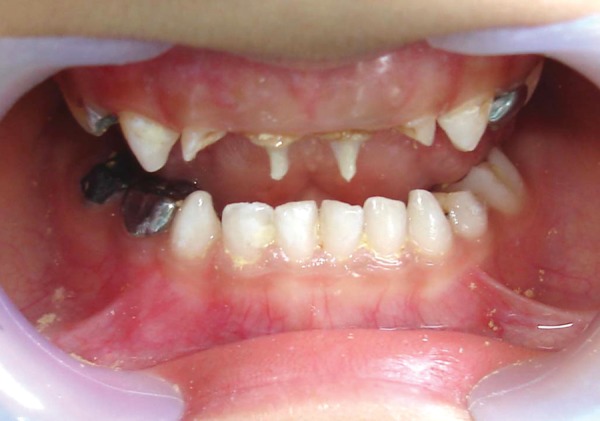
Fiberglass postinsertion (case 1)

**Fig. 4:**
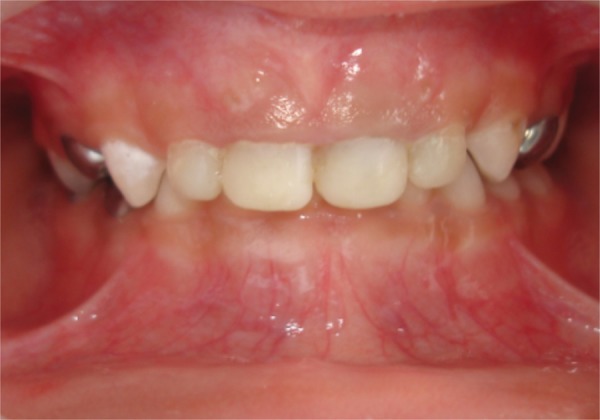
Postoperative anterior view

For each canal a post of corresponding size is trial fit for proper fitting and proper length. The post was placed to a distance of 3 mm into the canal and the length was adjusted, such that it extends 2 mm outside the canal. An intraoral radiograph was taken to ensure that the end of the post was at the level of interdental crest or just apical to it. Any excessive length of the post was cut with a diamond bur under water coolant. Then the prepared cavity was acid etched for 15 seconds with a 37% phosphoric acid gel, rinsed, dried and two coats of a dentin adhesive single bond (3M) was applied according to the manufacturer's instruction. The tip of flowable composites tube was placed 2 to 3 mm below the CEJ and the composite was injected. The glass post was inserted into the canal with cotton pliers. It was then light cured according to the manufacturer's instruction (Fig. 3). The coronary portion of the fiber was completely restored using resin composite. After checking the occlusion and the removal of any interference, final finishing and polishing of the restoration was performed with composite polishing disks (Fig. 4).

### Case 2

A 4-year-old female child reported with pain on biting in upper anterior region and was repeatedly put on anti- inflammatory and analgesic medication since last 6 to 7 months. The procedure was followed as described earlier and the completed restoration is shown in Figures 7 to 10.

## DISCUSSION

The successful restoration of badly mutilated primary anterior teeth in preschool children is a challenging task. The high failure rate of such restoration is due to the insufficient tooth structure available to support them. In addition, the poor adhesion of the bonding agents to the enamel and dentine of primary teeth as compared to that of permanent teeth can compromise the final restoration.^7^

Different resin materials and techniques have been used for reinforcing root canals. The use of intracanal posts in endodontically treated teeth improves the retention of a definitive restoration. A custom made ‘omega wire extension' placed inside the root cavity and fixed with a composite resin is another alternative. Though it is easier and inexpensive technique but does not get an adequate adaptation to the canal wall, which may lead to radicular fracture on excessive masticatory forces.^7,10^

The biological posts use natural extracted teeth that are prepared in a post shape for cementation in the root canal. The natural crowns offer outstanding anatomy and esthetics but it needs the creation of a tooth bank and secure methods of sterilization and storage to ensure the safety of teeth.^2,3^

The posts made with composite resin present a satisfactory esthetic result, but they have the risk of losing the retention due to polymerization contraction.^2,5^

It was shown from the above cases that the application of fiberglass post on badly destroyed primary incisor is a valuable clinical procedure. These technique increases the surface area of tooth structure, i.e. intraradicularly, where adhesion of the bonding of tooth structure is enhanced.^11^ The use of fiberglass post together with flowable composite and bonding agent offers an alternative where all components are bonded together to form a firmly attached restoration unit. Again his technique utilizes the coronal portion of the root, which is the strongest part of the root to transmit any functional stresses and may add to success.^2^

In a 1 year follow study, Aly A Sharaf^9^ found that restoration carried out on grossly broken down primary incisors using fiberglass posts remain intact. Laboratory studies have also demonstrated that this technique significantly improved the fracture resistance of teeth. In another clinical study Priya Subramaniam^12^ et al compare fiberglass post with omega shaped stainless steel wire in primary maxillary anterior teeth. After 1 year, they found fiberglass posts showed better retention and marginal adaptation than omega shaped stainless steel wire.

**Fig. 5:**
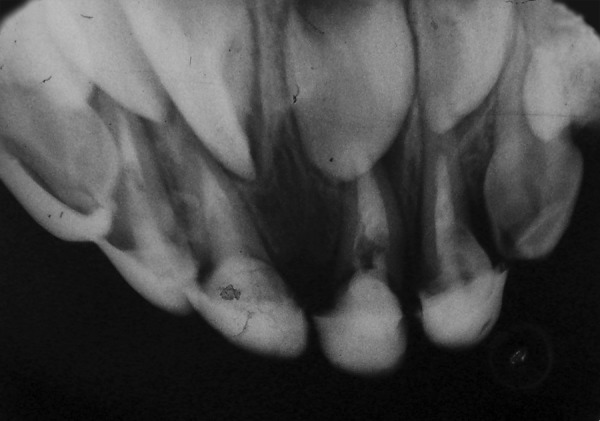
Radiograph after cementation of post

Radiographic evaluation after post placement is important to check the level of the post. In above cases, the posts were introduced inside the canals until the limit of the cervical third because, as described by Rifkin^13^ in 1983, a larger length may interfere with the eruption of the underlying permanent tooth during the final stages of resorption of the primary roots (Fig. 5). Also roots must be long enough to support the placement of post. Children 6 years or older would not have sufficient longevity and would not be candidates for this technique.

**Fig. 6:**
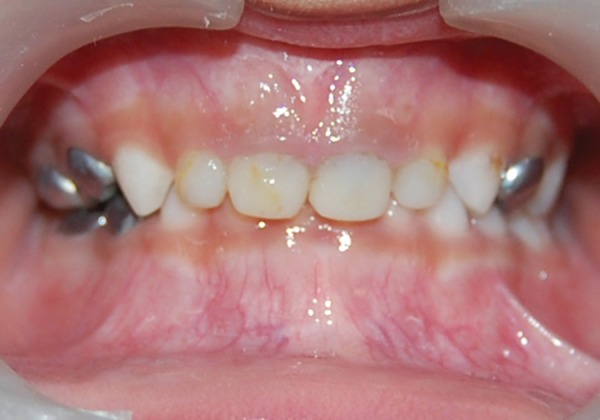
After 1 year (case 1)

In the presented clinical cases, it was possible to reconstruct all the upper anterior primary teeth using the glass fiber post. The 1 year recall appointments have revealed satisfactory clinical performance of the bonded restoration (Figs 6 and 10). Parents of both the children, who were treated, reported an improvement in psychological behavior of their children and felt that the lifestyle of the children had been improved by the restoration of their anterior teeth in the manner described. Since the method is technique sensitive, sometimes it may necessary to perform the procedure under sedation or general anesthesia considering the young age and cooperative ability of the patient. Periodic clinical and radiographic evaluation is essential together with parent cooperation regarding oral hygiene and dietary habits.

**Fig. 7:**
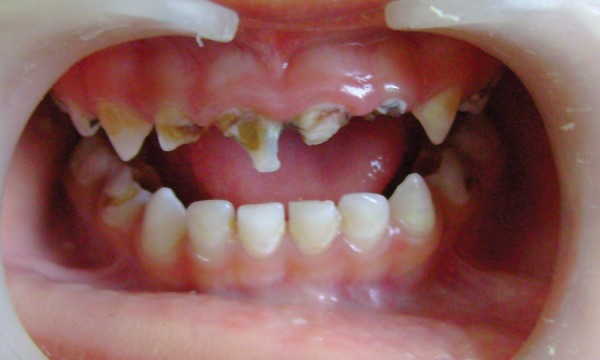
Preoperative (case 2)

## CONCLUSION

The discribed technique is simple and can be used to restore severly carious or no. of primary anterior teeth, reestablising function, shape and esthetic in young children. Glassfiber post as intracanal retention appears to be a valuable clinical procedure.

**Fig. 8:**
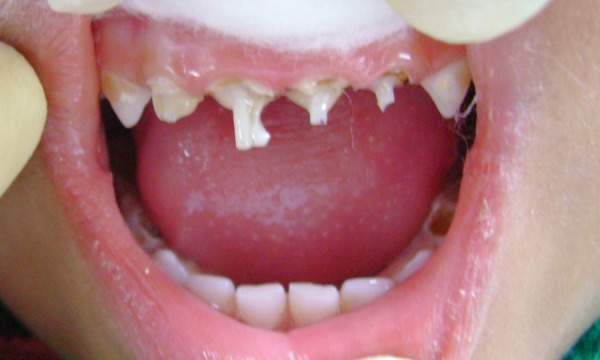
Fiberglass postinsertion (case 2)

**Fig. 9:**
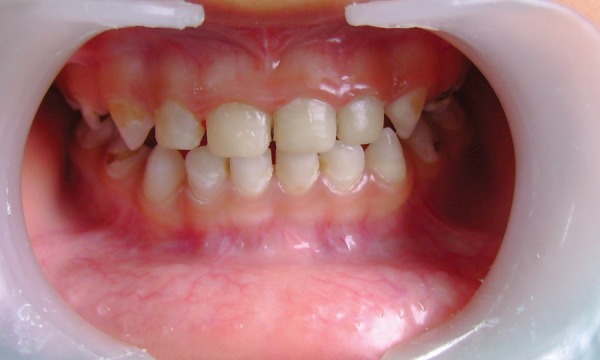
Immediate postoperative

**Fig. 10:**
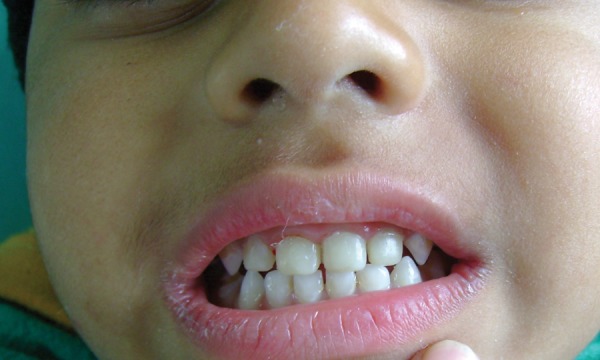
After 1 year (case 2)
